# Using novel natural language processing approaches to examine age-friendly communication about nursing Nome residents with dementia

**DOI:** 10.1093/geront/gnaf285

**Published:** 2025-12-04

**Authors:** Kimberly R Powell, Mira Isnainy, Suhwon Lee, Matthew S Farmer, Philip Amewudah, Mihail Popescu, Ashley Woods, Gregory L Alexander, David R Mehr

**Affiliations:** Sinclair School of Nursing, University of Missouri, Columbia, Missouri, 65211, United States; College of Arts and Sciences, University of Missouri, Columbia, Missouri, 65211, United States; College of Arts and Sciences, University of Missouri, Columbia, Missouri, 65211, United States; Sinclair School of Nursing, University of Missouri, Columbia, Missouri, 65211, United States; Institute for Data Science and Informatics, University of Missouri, Columbia, Missouri, 65211, United States; Department of Biomedical Informatics, Biostatistics, and Medical Epidemiology, University of Missouri, Columbia, Missouri, 65211, United States; Sinclair School of Nursing, University of Missouri, Columbia, Missouri, 65211, United States; School of Nursing, Columbia University, New York, New York, 10027, United States; Department of Family and Community Medicine, University of Missouri, Columbia, Missouri, 65211, United States

**Keywords:** Avoidable transfer, 4M framework, Text messages, Nursing informatics

## Abstract

**Background and Objectives:**

The Age-Friendly Health Systems model has emerged as a geriatric care model focusing on the 4Ms: what matters, mobility, mentation, and medications. Representation of the 4Ms in interdisciplinary communication could have implications for outcomes including avoidable nursing home-to-hospital transfers. The objective of this article was to explore the association between the 4Ms found in text messages and avoidable transfer of residents with and without Alzheimer’s Disease and Related Dementias (ADRD).

**Research Design and Methods:**

The researchers merged data from two primary datasets: (a) text messages (*n* = 30,066) between nursing home (NH) healthcare workers from an HIPAA-compliant messaging platform and (b) resident transfer data (*n* = 3,687) from NHs (*n* = 16) that participated in a 5-year (2016–2020) Centers for Medicare and Medicaid Services demonstration project. They used natural language processing to extract 4M terms from text messages and fit generalized linear mixed models to estimate avoidable NH-to-hospital transfer, controlling for resident characteristics, NH characteristics, and the 4Ms.

**Results:**

Merged data contained 1,031 observations grouped by temporal proximity to the transfer date. Cardiopulmonary resuscitation status, late-stage ADRD, NH bed size, and location were associated with avoidable NH-to-hospital transfer of residents. Text messages containing terms representing mentation and mobility were also associated with avoidable NH-to-hospital transfers.

**Discussion and Implications:**

These results suggest that natural language processing can be used to identify components of age-friendly care in unstructured data. Association between resident level and nursing home factors and avoidable transfers should be considered as nursing homes implement strategies to reduce avoidable transfer of residents with ADRD.

As the global population ages, there has been a notable rise in the number of individuals living with Alzheimer’s disease or related dementias (ADRD). A recent article published in *Nature Medicine* estimates the lifetime risk of developing ADRD after age 55 years is 42%, translating to approximately one million new cases per year by the year 2060 (Fang et al., 2025). This shift has significant implications for healthcare systems worldwide, particularly in nursing homes (NHs), where approximately 48% of 1.3 million residents have ADRD (Alzheimer’s Association, 2021).

Along with the increase in the ADRD population, advancements in technology, diagnostic methods, and treatment options have expanded dramatically. These innovations have not only improved clinical outcomes but have also prompted the development of new care models aimed at enhancing quality of life for older adults. This evolution underscores the necessity for healthcare systems to adapt in response to the growing demands of an aging population with increasingly complex care needs. Recognizing the need for a more integrated and person-centered approach to care of older adults, the Age-Friendly Health Systems (AFHS) model emerged as a proactive response (Institute for Healthcare Improvement [IHI], 2022). The premise of the AFHS initiative is to identify core issues that should drive decision-making with older adults and to shift care, on an organizational level, by centering care on the 4Ms: what matters, mobility, mentation, and medications (see Figure 1) (Institute for Healthcare Improvement, 2022). The framework prioritizes individual needs and preferences of older adults, aiming to optimize health outcomes and minimize unnecessary interventions including avoidable NH-to-hospital transfers.

**Figure 1.**
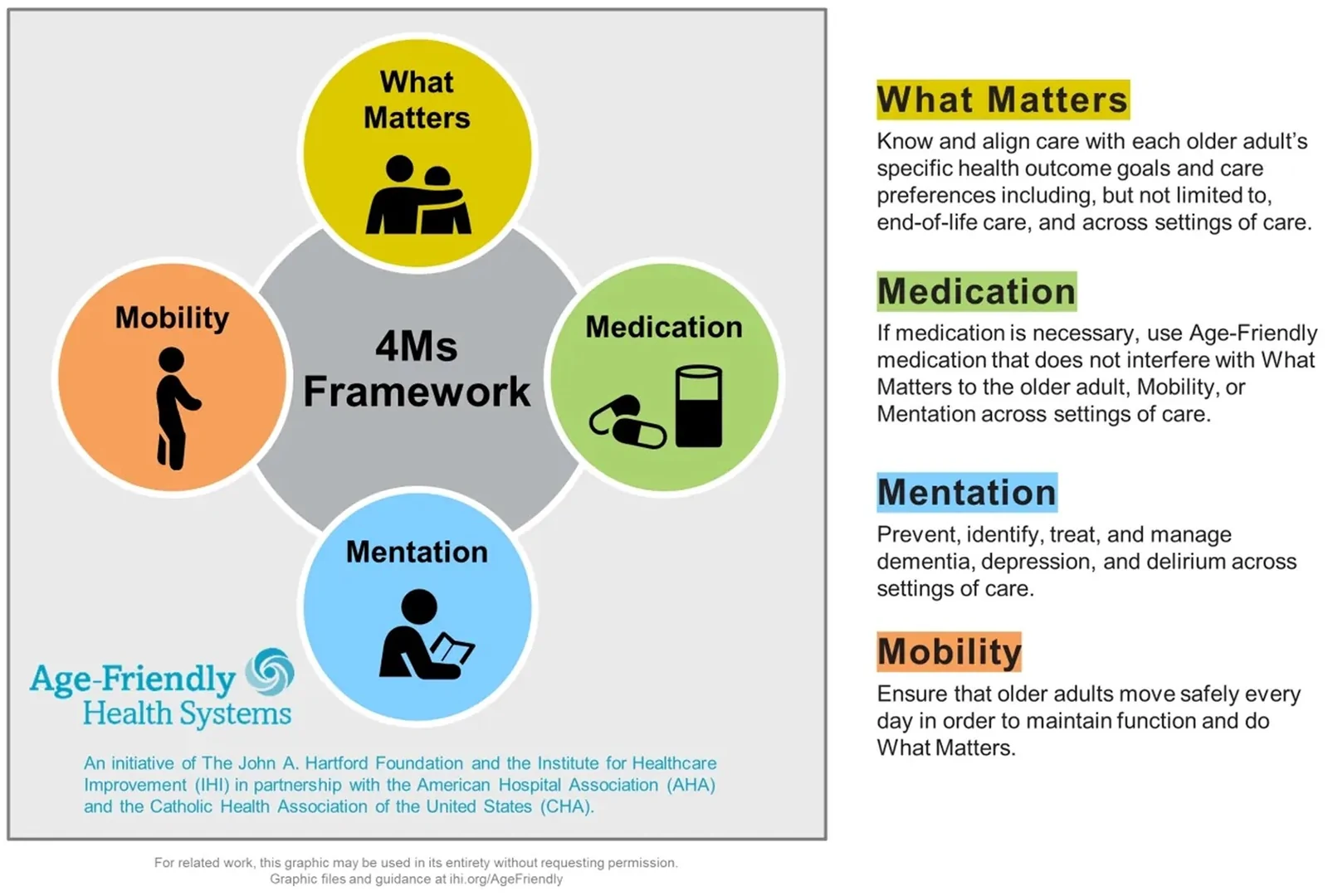
4Ms framework of an age-friendly health system.

**Figure 2.**
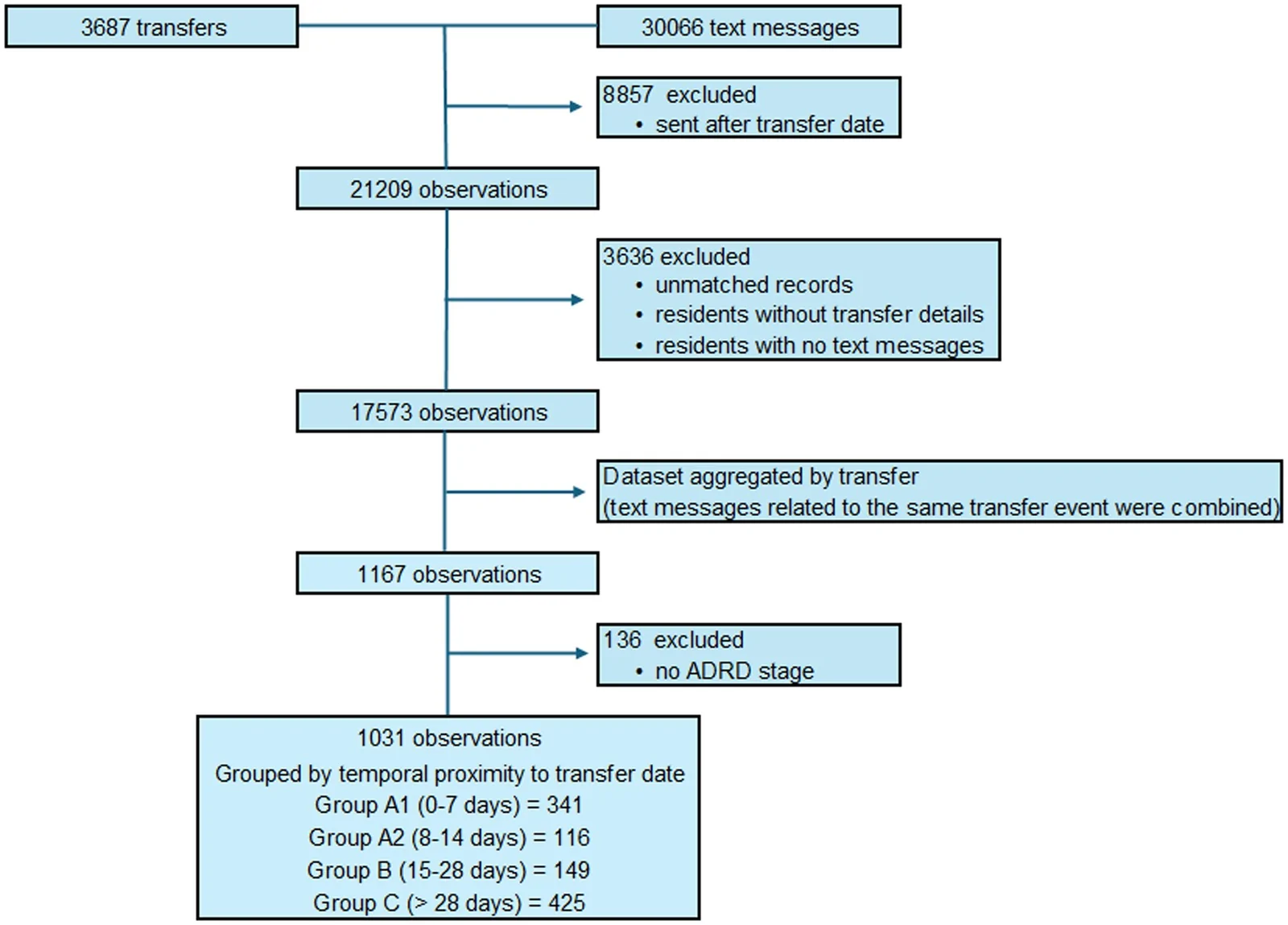
A flowchart illustrates how transfer records and text messages were combined and filtered to create the final analytic dataset. Starting with 3,687 transfers and 30,066 text messages, several exclusion steps removed messages sent after transfer dates, unmatched records, residents without transfer details, and those without ADRD stage information. The final dataset includes 1,031 observations grouped by how many days before the transfer the messages were sent. ADRD = Alzheimer’s disease or related dementias.

The 4M framework has theoretical linkages to minimizing unnecessary interventions for older adults in several ways. First, the central concept of the framework, what matters, ensures that care decisions are guided by each individual’s personal goals and preferences. This often means prioritizing comfort, dignity, or remaining in place rather than pursuing burdensome hospitalizations that might negatively impact quality of life. Negative effects of hospital transfer, especially for residents with cognitive impairment, are well documented in the literature and include physical and psychological decline, increased risk of infection, and disruptions in continuity of care (Powell et al., 2024; Ryman et al., 2019). Second, the focus on medications could help mitigate long-standing problems such as inappropriate prescribing and adverse drug events, both of which contribute to avoidable hospitalizations (Boockvar et al., 2004; Rapp et al., 2023). Finally, the concepts of mentation and mobility represent two common drivers of avoidable NH-to-hospital transfers: cognitive changes and falls (Allan et al., 2009; Hshieh et al., 2020). Collectively, the 4M framework offers a structured, person-centered approach to care that addresses many common contributors to avoidable NH-to-hospital transfers. Incorporating these elements into interdisciplinary communication could help healthcare teams make timely, evidence-based decisions that align with resident goals and values.

Healthcare team communication is a key driver of patient outcomes. The link between poor interprofessional communication within healthcare teams and adverse patient outcomes is well established (Greenberg et al., 2007; Risser et al., 1999; Sutcliffe et al., 2004). The science of healthcare team communication has emerged as an important field in the 21st century as initiatives to support healthcare teamwork have grown and more attention is being paid to clinician well-being and strategies to mitigate burnout (Donohue-Ryan et al., 2023; Rossetti et al., 2024). Text messaging has become a convenient, low-cost communication tool used by healthcare teams. Recent studies examining the use of text messaging applications have focused on communication taking place in laboratory departments (Dorwal et al., 2016), consultations with external providers (Astarcioglu et al., 2015; Gulacti et al., 2016), and between members of a surgical team (Johnston et al., 2015). While numerous interventions (Ouslander et al., 2014, 2010) and demonstration projects (Blackburn et al., 2021; Rantz et al., 2017, 2021) have aimed to reduce avoidable transfers, no studies to our knowledge have examined the impact of communication, aligned with the 4Ms, on avoidable NH-to-hospital transfers. The objective of this article was to explore the association between the 4Ms found in text messages exchanged among the interdisciplinary care team and avoidable transfer of residents with and without ADRD.

## Methods

### Sampling and setting

This cohort article used two primary datasets: (a) text messages between NH healthcare workers from an HIPAA-compliant messaging platform called Mediprocity and (b) transfer data from the Missouri Quality Initiative (MOQI), a 5-year (2016–2020) Centers for Medicare and Medicaid Services (CMS) demonstration project (1E1CMS331080). NHs (*n* = 16) participating in the MOQI project had access to similar resources including (a) advanced practice registered nurses (APRNs) working in each NH, providing support to frontline clinicians, (b) feedback reports about hospitalizations and transfers, (c) use of Interventions to Reduce Acute Care Transfers (INTERACT) tools (Ouslander et al., 2014), and (d) access to enhanced communication systems (e.g., secure text messaging) to facilitate health information exchange (Rantz et al., 2017). A comparison of the MOQI NHs to the overall U.S. NH population is provided in the online supplementary material. Compared to other regions, the Midwest has the largest number of NH beds per 1,000 persons age 65 and older. The most common diagnoses for NH residents in our sample (with an NH-to-hospital transfer) were ADRD, stroke, chronic kidney disease, heart failure, and chronic obstructive pulmonary disease. These same diagnoses represent 5 of the 10 most ­common diagnoses among all U.S. NH residents (Centers for Medicare and Medicaid Services, 2012).

Each time a resident was transferred from the NH to the hospital, regardless of the outcome of the transfer (e.g., emergency department visit only, hospital admission, etc.), APRNs completed a Qualtrics survey based on the INTERACT tool. Broad categories of the survey included resident characteristics, clinical and nonclinical factors contributing to the transfer, and actions taken to evaluate and manage the condition before transfer. NHs that participated in the project ranged in bed size from 120 to 300 beds, and were in metropolitan, micropolitan, and rural settings (see Table 1). Residents enrolled in the MOQI project were dual eligible for Medicare and Medicaid services. Short-stay residents, defined as those residing in the NH for more than 100 days, were excluded from the demonstration project. For this analysis, text messages sent in close temporal proximity to the transfer event were linked to transfer events recorded in the MOQI survey.

**Table 1.** Sample demographics.

Parameter	(*n* = 378 unique residents)	
	*n*	%
**Age**		
** ≤64**	94	24.87
** 65–74**	101	26.72
** 75–84**	91	24.07
** ≥85**	92	24.34
**Sex**		
** Female**	236	62.43
** Male**	142	37.57
**Race**		
** Black or African American**	73	19.31
** White**	302	79.89
** Other**	3	0.79
**CPR status**		
** Full code**	181	48.01
** Do not resuscitate**	182	48.28
** Other**	14	3.71
**ADRD status**		
** No ADRD**	210	55.56
** Early**	22	5.82
** Middle**	84	22.22
** Late**	62	16.4

*Note.* ADRD = Alzheimer’s disease or related dementias; CPR = cardiopulmonary resuscitation.

### Ethics approval

This study was approved by the University of Missouri Institutional Review Board (2009109).

### Text extraction

Prior to natural language processing (NLP) of the text messages, a “gold standard” dataset was annotated by two clinical experts familiar with the 4M framework to serve as evaluation for NLP techniques developed by the research team. The gold standard dataset included a sample of 860 messages from a random sampling of 40 transfer events. Two clinical experts reviewed each text message, independently extracted words or phrases that aligned with one of the 4Ms and met to discuss and align any differing extractions. Two NLP techniques (supervised machine learning and large language model) were used to extract 4M content from text messages exchanged between NH care teams. Supervised machine learning relies on predefined rules and labeled training data to identify specific terms in narrative notes, whereas a fine-tuned large language model leverages broader language understanding. Both techniques have been used in other studies to successfully extract meaningful clinical data from free-text (narrative) sources (Khademi et al., 2025; Kim et al., 2025).

#### Supervised machine learning approach

To support automated concept recognition, a machine learning or 4M-Entity Extraction (4M-EE) pipeline was developed to map text to relevant 4M concepts using semantic representation through term embeddings. The pipeline consisted of two stages, first, classifying clinical text at the message level and then again at the key phrase level to enhance precision. The researchers introduced and evaluated a cosine-distance based possibilistic K-nearest neighbors’ (PKNN) model, a variation of the traditional K-nearest neighbor (KNN) algorithm. Traditional KNN models classify data by taking a majority vote from the closest data points. In contrast, the PKNN approach considers both the majority vote and the degree of uncertainty that comes from how far the data points (i.e., vectors) are from each other, while also using a measure of similarity (cosine distance) that emphasizes direction rather than size (Lin et al., 2014). The researchers compared this model to baseline machine learning models and to a fine-tuned Bidirectional Encoder Representation from Transformers model in the pipeline (Li et al., 2019). For example, in the message “resident was alert and oriented,” the term “alert” is represented as a vector using term embeddings that capture its semantic meaning. This embedding is then compared to vectors representing predefined 4M concepts, enabling the system to recognize that “alert” aligns closely with mentation. This approach allows the pipeline to identify relevant concepts even when different but semantically similar terms are used.

#### Large language model approach

A generative large language model (LLM), Gemma 2 9 b (9 billion parameters), was fine-tuned on a subset of the gold standard dataset and coded to extract individual words and phrases that aligned with 4M content. The LLM was locally hosted to ensure that all data processing occurred within secure institutional servers, thereby protecting resident privacy and maintaining compliance with data security standards. These extractions were further utilized to create a taxonomy of 4M concepts and related terms. Development and evaluation of the 4M taxonomy, including robust descriptions of the text messages, has been previously published elsewhere (Farmer et al., 2025).

### Natural language processing validation

The supervised machine learning and LLM extractions were performed using Python with Transformers package. NLP extractions were compared to the gold standard dataset using classification and count comparisons to assess if the different techniques were extracting the same amount of 4M content from the same messages using metrics including F1 score, Precision, Recall, Cohen’s Kappa, and mean absolute error.

### Data cleaning and processing

The data cleaning and processing stage aimed to ensure consistency and accuracy in the dataset used for analysis. To make relevant comparisons between ADRD and other diagnoses, the necessary variables were imported into the text message dataset. The merging process between these two datasets resulted in a dataset containing 21,209 observations.

As many residents had multiple transfers, it was necessary to determine the corresponding text messages to the nearest transfer date. The text messages were categorized into four groups based on their temporal proximity to the transfer date: Group A1 (0–7 days before), Group A2 (8–14 days before), Group B (15–28 days before), and Group C (more than 28 days before transfer). After aggregating text messages by transfer date for each patient, the dataset was reduced to 1,031 observations (Figure 2).

### Statistical analysis

The researchers performed the analysis twice: using all groups (A1, A2, B, and C) and using only group A, that is, messages exchanged 0–14 days before transfer. While there is some evidence demonstrating that technology can facilitate detection of declining conditions in older adults one to two weeks before an event occurs (Rantz et al., 2012), the longitudinal nature of NH care, presence of multiple chronic conditions among NH residents, and complex care needs warrant a broader scope to evaluate outcomes and the effectiveness of care interventions. All statistical analyses were performed using RStudio.

#### Variables

Determination of the outcome measure (avoidable NH-to-hospital transfer) was made by a team consisting of the APRN, project coordinator, and APRN supervisor. Using an iterative interrogative approach, the team explored the root causes of each transfer using the quality improvement technique “Five Why’s” (Institute for Healthcare Improvement, 2018). After exploring cause and effect to assure root causes of transfers were considered, a consensus was met on which transfers were deemed avoidable versus unavoidable (Vogelsmeier et al., 2021). In the primary study, data were collected only for residents who were transferred to the hospital; therefore, unavoidable transfers were used as the comparison group. The explanatory variables included demographic and clinical characteristics including age, sex, race, transfer date groups, ADRD stage (based on Alzheimer’s Association (2024) definitions), cardiopulmonary resuscitation (CPR) status, NH bed size, location, and extracted 4M components (What Matters, Medication, Mentation, Mobility). Explanatory variables were selected based on prior work and data availability. For example, in prior work, NH location has been found to be a significant predictor of resident outcomes and NH quality measures (Temkin-Greener et al., 2012).

#### Generalized linear mixed models

Due to the hierarchical nature of the data, where individual patient records are nested within NHs, the researchers fit Generalized Linear Mixed Models (GLMMs). GLMM addresses correlations within groups by incorporating random effects to capture variations specific to each NH, alongside fixed effects that estimate population-level trends. This dual-level modeling ensures that standard errors are properly adjusted, reducing the risk of underestimation and false positives (Molnar, 2020). The researchers applied an optimization technique during model fitting to ensure numerical stability and reliable parameter estimation. This method allows the model to efficiently handle complex likelihood functions and prevent convergence issues. By setting appropriate control parameters, the estimation process ensures that the model reaches a well-optimized solution without excessive computational burden.

The analysis consisted of two approaches for counting 4Ms: one using count data and another using normalized data. Normalization was performed using min–max normalization, which scales the data between 0 and 1. Using both count data and normalized data allows for a comprehensive assessment of model performance under different preprocessing approaches.

#### Model fit and selection

To evaluate model performance, the Akaike Information Criterion (AIC) was used as the primary model selection criterion, with lower AIC values indicating a better-fitting model. AIC is particularly useful in model comparison because it penalizes overly complex models while rewarding good predictive accuracy (Akaike, 1974). This ensures that the chosen model is not just accurate but also generalizable to new data. In this study, the lowest AIC value highlighted the model’s effectiveness in capturing relevant relationships without overfitting. As multiple model configurations were tested, the analysis compared four settings: (a) count data (of extracted 4M components) using the supervised machine learning approach, (b) normalized data (of extracted 4M components) using the supervised machine learning approach, (c) count data extractions using the LLM approach, and (d) normalized data extractions using the LLM approach. The model with the lowest AIC was selected to report in the results. All models are included in the online supplemental material.

## Results

### Descriptive statistics

The article sample included 378 unique residents. Most residents were female (62.4%) and white (79.9%); 48.3% had CPR status of do not resuscitate, and the mean (SD) age was 73.1 (13.61) years (Table 1). The number of transfer events used in this analysis was 1,031 of which 639 (62%) were unavoidable and 392 (38%) were avoidable.

### Generalized linear mixed model results

Among the four models considered, the GLMM model using the supervised machine learning approach to extract 4M concepts with count data for residents with ADRD had the lowest AIC (896.03), suggesting this model provided the best balance between model fit and complexity. This indicates that using count data rather than normalized data for residents with ADRD led to better predictive accuracy of avoidable/unavoidable transfer while avoiding unnecessary model complexity. The selection of this model highlights the importance of preserving the original data distribution for ADRD-related transfers, as normalization may reduce the interpretability of key factors influencing avoidable transfers.

Table 2 depicts the GLMM output using all groups with random effects where individual transfers are nested within NHs. Residents with late-stage ADRD were significantly more likely to have an avoidable NH-to-hospital transfer (OR 16.03; 95% CI, 2.19, 117.39; *P*=.01) compared to residents without ADRD. Residents with CPR status of full code (OR 0.17; 95% CI 0.06, 0.51; *P*=.001) and “other” (OR 0.001; 95% CI, 0.00, 0.09; *P*=.002) were less likely to have an avoidable NH-to-hospital transfer compared to those with a “do not resuscitate” order. NH factors including bed size of 160–220 beds compared to bed size of more than 160 (OR 0.01; 95% CI, 0.00, 0.24; *P*=.01) and rural location compared to metro area (OR 0.0008; 95% CI, 0.00, 0.05; *P*=.001) were associated with fewer avoidable NH-to-hospital transfers. Among the 4M concepts, mentation was the only statistically significant variable associated with avoidable transfer (OR 0.91; 95% CI, 0.82, 1.00; *P*=.05). Table 3 presents the GLMM output using group A only, that is, messages exchanged within 14 days of transfer. When healthcare teams included terms representing the 4M concept of mobility in text messages, residents with ADRD were more likely to have an avoidable NH-to-hospital transfer (OR 1.21; 95% CI, 1.02, 1.42; *P *= 0.03). While some other variables had odds ratios suggesting a substantial relationship with this restricted model (e.g., 3.68 for late stage ADRD or 0.15 for bed size of 160–220), the confidence intervals all included 1.

**Table 2.** Generalized linear mixed models output for all groups.^a^

Parameter	OR (95% CI)
**Intercept**	0.62 (0.00–110.68)
**Age group**	
** ≤64**	1 [Reference]
** 65–74**	4.74 (0.93–24.16)
** 75–84**	2.96 (0.52–16.87)
** ≥85**	6.26 (0.85–46.11)
**Race**	
** Other**	1 [Reference]
** Black or African American**	5.70 (0.05–677.80)
** White**	4.03 (0.04–443.38)
**Sex**	
** Female**	1 [Reference]
** Male**	2.00 (0.56–7.19)
**ADRD status**	
** No (reference)**	1 [Reference]
** Early**	0.16 (0.01–1.90)
** Middle**	0.77 (0.17–3.58)
** Late**	16.03 (2.19–117.39)*
**CPR status**	
** Do not resuscitate**	1 [Reference]
** Full code**	0.17 (0.06–0.51)*
** Other**	0.00 (0.00-0.09)*
**Bed size**	
** <160**	1 [Reference]
** 160–220**	0.01 (0.00–0.24)*
** >220**	0.11 (0.01–1.09)
**Location**	
** Metropolitan**	1 [Reference]
** Micro**	2.11 (0.15–30.44)
** Rural**	0.00 (0.00–0.05)*
**Groups^b^**	
** A1 (0–7 days)**	1 [Reference]
** A2 (7–14 days)**	0.67 (0.26–1.73)
** B (15–28 days)**	0.74 (0.33–1.67)
** C (more than 28 days)**	1.13 (0.61–2.11)
**4M terms**	
** What Matters**	1.05 (0.98–1.12)
** Mentation**	0.91 (0.82–1.00)*
** Medication**	1.02 (0.99–1.06)
** Mobility**	0.95 (0.86–1.04)

*Note*. ADRD = Alzheimer’s disease and related dementias; CPR = Cardiopulmonary resuscitation; NH = Nursing Home; OR = Odds ratio.

a All messages sent prior to transfer were included in this model.

b Groups indicate the number of days the text message was sent prior to the NH-to-hospital transfer.

* *P* < .05.

**Table 3.** Generalized linear mixed models output for group A only.^a^

Parameter	OR (95% CI)
**Intercept**	0.82 (0.09–7.49)
**Age Group**	
** ≤64**	1 [Reference]
** 65–74**	1.4 (0.43–4.52)
** 75–84**	1.33 (0.38–4.64)
** ≥85**	0.97 (0.21–4.45)
**Race**	
** Black or African American**	1 [Reference]
** White**	1.01 (0.24–4.32)
**Sex**	
** Female**	1 [Reference]
** Male**	1.29 (0.52–3.2)
**ADRD status**	
** No**	1 [Reference]
** Early**	0.28 (0.03–2.55)
** Middle**	0.49 (0.15–1.61)
** Late**	3.68 (0.78–17.33)
**Code status**	
** Do not resuscitate**	1 [Reference]
** Full code**	0.44 (0.18–1.1)
** Other**	0.08 (0–4.25)
**Bedsize**	
** <160**	1 [Reference]
** 160–220**	0.15 (0.01–2.8)
** >220**	1.41 (0.19–10.67)
**Location**	
** Metropolitan**	1 [Reference]
** Micro**	3.46 (0.28–42.73)
** Rural**	1.5 (0.01–161.33)
**4M terms**	
** What matters**	0.95 (0.86–1.05)
** Mentation**	0.95 (0.82, 1.11)
** Medication**	0.98 (0.93–1.04)
** Mobility**	1.21 (1.02–1.42)*

*Note*. ADRD = Alzheimer’s disease and related dementias; OR = Odds ratio.

a Group A included messages sent 14 days or less before transfer.

* *P* < .05.

## Discussion

To the researchers’ knowledge, this article is the first to examine the associations between 4M content found in text messages and avoidable NH-to-hospital transfer of residents with ADRD. Using the 4M-EE pipeline, the researchers were able to extract and compare 4M content from all text messages and text messages exchanged within two weeks of transfer. CPR status (full code and other), late-stage ADRD, facility bed size and location (rural) were associated with avoidable NH-to-hospital transfer of residents. Text messages about mentation were associated with fewer avoidable NH-to-hospital transfers. When messages were limited to those sent within 14 days of transfer, mobility was significantly associated with increased avoidable NH-to-hospital transfers.

### Implications for research

The researchers demonstrated that NLP can be used to identify components of age-friendly care, that is, the 4Ms in unstructured data. The sequelae of neurodegenerative conditions like dementia include loss of language, complicating the decision-making process regarding the need/desire to transfer the resident to the hospital. As these individuals gradually lose the ability to express their needs, preferences, and symptoms verbally, valuable insights about their condition and care goals can be hidden in unstructured text created by clinicians, caregivers, and family members. Mining these data can reveal patterns of behavioral changes, unmet needs, or early signs of decline that may otherwise go unnoticed. Importantly, these insights can inform more responsive, person-centered interventions and care planning. Future studies should consider additional data sources and representations of care aligned with the AFHS 4M framework.

Several resident-level factors were significantly associated with the odds of experiencing an avoidable nursing home-to-­hospital transfer. Residents with a CPR status of “Full Code” or “Other” had significantly lower odds avoidable transfers compared to those with a “DNR” status (OR = 0.17 and OR = 0.0014, respectively). This finding suggests that resuscitation preferences may meaningfully influence decisions about hospital care, even when the transfer is considered avoidable. While it might seem counterintuitive that residents with Full Code status, who presumably desire more aggressive treatment, have lower odds of avoidable transfer, this may reflect clinical decision-making processes where DNR status is used not only to guide code events but also as a proxy for broader goals of care.

Similar findings have been reported in the literature suggesting that DNR orders are often associated with higher rates of potentially avoidable transfers, particularly when there is uncertainty about the residents’ treatment preferences beyond resuscitation (Ernecoff et al., 2018; Hanson et al., 2017). For example, in a cluster randomized clinical trial, Cohen et al. (2019) found that although most nursing home residents with advanced dementia had directives to withhold aggressive interventions such as CPR, it was far less common to see documentation addressing less invasive treatments like antibiotics or intravenous fluids. This suggests that CPR status may reflect a limited scope of planning, leaving other treatment decisions open to interpretation. Further research is needed to explore how resuscitation preferences influence transfer decisions and how the clarity and comprehensiveness of advanced care planning documentation reflects the goals and preferences of residents and their families.

The researchers found that residents from NHs located in rural areas and those with bed size of 160–220 were associated with lower odds of avoidable NH-to-hospital transfer. This finding is consistent with others who have reported lower rehospitalization rates among skilled nursing facilities located in rural areas compared to those located in urban areas (Clement et al., 2018). Lower transfer rates in rural areas may reflect the influence of rural culture (Baernholdt et al., 2010). For example, challenges like geographic isolation, severe weather, and limited access to services may foster a heightened sense of community connectedness (Clement et al., 2018). Given that nursing homes located in rural areas often operate with limited resources, including fewer on-site clinical staff, reduced access to specialized services, and less robust infrastructure to manage acute changes in condition, exploring the relationship between resource availability, access to specialized care, and overall care quality is critical. These relationships should be the focus of future inquiry especially given the proposed changes to Medicaid reimbursement and new policy initiatives (i.e., Rural Transformation Fund) aiming to support rural hospitals and other health care providers in the face of potential funding cuts (National Rural Health Association, 2025).

Notably, terms related to “what matters” and “medications” were not significantly associated with avoidable NH-to-hospital transfers in this study. Several factors may help explain these null findings. First, documentation of resident goals and preferences may not be as common in text messages as in other forms of clinical documentation. Even when present, extracted terms may not reflect the granularity needed to detect relationships with avoidable transfer, such as distinctions between specific care goals. The lack of association between medications and avoidable NH-to-hospital transfers may reflect the routine nature of medication changes in NHs. Adjustments to medications are common and often occur in response to clinical changes, reducing the likelihood that a single medication issue directly precipitates a transfer. Further research should explore these concepts in additional data sources (including the electronic medical record) and explore the timing and context of their occurrence to better understand their relationship to avoidable NH-to-hospital transfers.

### Implications for practice and policy

The researchers found that when NH care teams were communicating about mentation (i.e., for each additional mention of mentation), the odds of avoidable transfer decreased by 9%. The significance of this predictor variable in our dataset, including all messages, implies that communication about mentation is taking place well in advance (more than two weeks) of the transfer event. Differentiating acute delirium from ADRD has been the focus of much work due to poor outcomes associated with acute delirium and overlapping symptoms of delirium and dementia (Fong et al., 2022). Variability in clinical presentation of different types and stages of ADRD further complicates differentiating an acute from a chronic condition. For example, older adults with dementia due to Lewy body disease, may have fluctuating symptoms, further complicating the clinician’s ability to detect potential changes due to delirium; others with moderate or severe ADRD experience late-day increases in disorientation and agitation, referred to as “sundowning syndrome” (Taylor et al., 2020; Toccaceli Blasi et al., 2023). At the heart of all screening tools for delirium is the ability to recognize subtle changes in mentation, requiring familiarity with the residents’ baseline (Fick, 2022). Because of this, addressing the staffing crisis in U.S. NHs, where turnover rates are twice that of hospitals (Dean et al., 2023), is paramount for improving outcomes. High turnover not only disrupts continuity of care but also contributes to avoidable NH-to-hospital transfers, highlighting the urgent need for strategies to enhance staff retention and promote consistent caregiving.

Our findings suggest that increasing the frequency of communication about changes in mentation could lead to reduced avoidable NH-to-hospital transfer. Poor interdisciplinary communication has been reported as a contributing factor to avoidable NH-to-hospital transfer (Powell et al., 2024; Vogelsmeier et al., 2022). Clearly communicating about behavioral symptoms is critical for early detection of cognitive decline and timely intervention. Given that the majority of NHs rely on antiquated communication tools like fax and phone (Powell et al., 2021), using modern, low-cost technologies like text messaging could help mitigate barriers including difficulty contacting an on-call provider, especially during off peak times such as evenings and weekends (Longo et al., 2002).

When the researchers limited the analysis to text messages exchanged within two weeks of the transfer event, they found that when mobility is mentioned the odds of avoidable transfer increased by 21%. When they considered the terms representing the concept of mobility in text messages, resident falls was a frequent topic of discussion. Terms representing mobility were extracted from 511 transfers in the sample, of which 107 (21%) included the word “fall” or “fell.” Approximately half of all NH residents fall annually and one in three will fall two or more times in a year (AHRQ, 2017). When a resident falls in an NH, the immediate concern is ensuring their well-being and determining the extent of any injuries. While it is alarming that 9%–15% of resident falls lead to serious injury, it is reassuring that the majority do not sustain serious harm (Hendrich et al., 2003). This underscores the importance of thorough assessment in situ, without immediate hospital transfer. Staff training on interventions to mitigate falls and post-fall assessment is essential to ensure NH staff can accurately identify signs of injury and determine the appropriate level of care. Well-trained staff are more confident and consistent in their evaluations, leading to better decision-making about whether a resident can be safely managed in place or requires hospital transfer.

### Strengths and limitations

This study has notable strengths. First, the researchers successfully used NLP to extract 4M terms from text messages exchanged among the interdisciplinary healthcare team. They experimented with multiple NLP approaches, leveraging machine learning and a fine-tuned LLM, to enhance robustness in the approach. Second, using the AFHS 4M framework as our conceptual foundation is a strength because it provides a well-established, evidence-based structure prioritizing the needs of older adults. Grounding research in this framework ensures alignment with national quality improvement efforts and allows for clear connections to be made between study outcomes and real-world practice. In addition, using the 4M framework facilitates the translation and dissemination of findings to health systems already engaged in age-friendly initiatives, increasing the potential for practical impact and adoption.

This article also has limitations that must be acknowledged. First, data in this study were from NHs participating in the MOQI study and were all located in the state of Missouri. Second, the researchers extracted 4M terms from one data source: text messages exchanged among the interdisciplinary care team. Future studies should explore communication about the 4Ms in additional sources such as the electronic medical record. Third, this analysis was limited to residents who had an NH-to-hospital transfer and those with dementia stage documented in the transfer dataset. This limitation arises from the filtering process during data cleaning, which excluded patients without such staging, potentially biasing the results toward those with more complete records. Finally, while the researchers used multiple NLP approaches (machine learning and LLM) to enhance the robustness of 4M extraction from text messages, variability in clinical language, ambiguity, and context dependent meanings can result in inaccurate or incomplete extractions.

## Conclusions

This article has important implications for NH residents, staff, and providers. Factors associated with avoidable NH-to-hospital transfer among residents included CPR status, late stage ADRD, NHs with 160–220 beds and those located in rural areas. Text messages containing terms related to mentation were also significant when the researchers included all messages in the dataset. When the analysis was limited to messages exchanged in close temporal proximity to the transfer (<2 weeks), text messages containing terms related to mobility were statistically significant. Future research should examine how incorporating the 4Ms, specifically mentation and mobility, into clinical decision making can reduce avoidable NH-to-hospital transfers.

## Supplementary Material

gnaf285_Supplementary_Data

## Data Availability

This study has not been preregistered. Data are not available at this time as the researcher has not completed planned or expected analyses for future publications.
