# Supplementary material for: Using novel natural language processing approaches to examine age-friendly communication about nursing Nome residents with dementia
Source: Gerontologist. 2025 Dec 4;66(3):gnaf285. doi: 10.1093/geront/gnaf285 (PMC13377231; doi:10.1093/geront/gnaf285)
Supplement: gnaf285_Supplementary_Data [file gnaf285_supplementary_data.docx]

**Using Novel Natural Language Processing Approaches to Examine Age-Friendly Communication about Nursing Nome Residents with Dementia**

**Kimberly R. Powell, PhD, RN^1*^, Mira Isnainy, MS^2^, Suhwon Lee, PhD^2^, Matthew S. Farmer, PhD, RN^1^, Philip Amewudah, PhD(c)^3^, Mihail Popescu, PhD^4^, Ashley Woods, PhD(c), RN^1^, Gregory L. Alexander, PhD, RN^5^, David R. Mehr, MD, MS^6^**

^1^Sinclair School of Nursing, University of Missouri, Columbia, MO 65211, USA

^2^College of Arts and Sciences, University of Missouri, Columbia, MO USA

^3^Institute for Data Science and Informatics, University of Missouri, Columbia, MO, USA

^4^Department of Biomedical Informatics, Biostatistics, and Medical Epidemiology, University of Missouri, Columbia, MO, USA

^5^School of Nursing, Columbia University, New York, NY, USA

^6^Department of Family and Community Medicine, University of Missouri, Columbia, MO, USA

Emails: [miraayuisnainy@missouri.edu](mailto:miraayuisnainy@missouri.edu) (Mira Isnainy), [leesuh@missouri.edu](mailto:leesuh@missouri.edu) (Suhwon Lee), [msfppy@missouri.edu](mailto:msfppy@missouri.edu) (Matthew S. Farmer), [pagd2@missouri.edu](mailto:pagd2@missouri.edu) (Philip Amewudah), [popescum@health.missouri.edu](mailto:popescum@health.missouri.edu) (Mihail Popescu), [awry8@missouri.edu](mailto:awry8@missouri.edu) (Ashley Woods), [ga2545@cumc.columbia.edu](mailto:ga2545@cumc.columbia.edu) (Gregory L. Alexander), [mehrd@health.missouri.edu](mailto:mehrd@health.missouri.edu) (David R. Mehr)

*Corresponding Author: [powellk@missouri.edu](mailto:powellk@missouri.edu) (Kimberly R. Powell); 914 Hitt Street, Columbia, MO, 65211, USA; Phone (502) 640-7556

Supplementary Materials

| **Supplemental Table 1. Comparison of MOQI NHs to all U.S. NHs (2016-2020)** | | |
| --- | --- | --- |
|  | MOQI NHs (n=16) | Total U.S. NHs (N=15,600) |
| Location  Metropolitan  Micropolitan  Rural | 10 (62%)  3 (19%)  3 (19%) | 11,154 (71%)  2,168 (14%)  2,277 (15%) |
| Ownership  Non-profit  For-profit | 2 (12%)  14 (88%) | 3,744 (24%)  11,865 (76%) |
| Average number of certified beds (mean) | 120-321 (198) | 2-1,389 (106) |
| % Medicaid-certified  % Medicare-certified | 16 (100%)  16 (100%) | 14,820 (95%)  15,288 (98%) |

| Supplemental Table 2. Example Text Messages and 4M Extractions | | | | |
| --- | --- | --- | --- | --- |
|  | Message | Expert Annotations | Supervised ML Extractions | LLM (Gemma 2) Extractions |
| 1. | Start sertraline 50 mg a day Restart Lasix Make Tylenol in the a.m. 650 mg routine and keep PRN order | [('sertraline 50 mg a day', 'medication'), ('restart Lasix', 'medication'), ('make tylenol in the a.m. 650 mg routine', 'medication'), (' keep prn order', 'medication')] | [('sertraline 50 mg a day', 'medication'), ('lasix', 'medication'), ('tylenol', 'medication'), ('650 mg routine', 'medication'), ('prn', 'medication')] | [('sertraline 50mg a day', 'medication'), ('lasix', 'medication'), ('Tylenol 650mg', 'medication')] |
| 2. | James says he wants dnr! He is going to sign when he is in next | [('wants dnr', 'what_matters')] | [('dnr', 'what_matters')] | [('DNR', 'what_matters'), ('sign', 'what_matters')] |
| 3. | Can you see you when you come and speak to the daughter. They are concerned because [resident] doesn't seem to be getting better. I'll have a them get some VS for you | [('daughter', 'what_matters')] | [('daughter', 'what_matters'), ('concerned', 'what_matters')] | [('daughter', 'what_matters'), ('concerned', 'what_matters')] |
| 4. | PT/INR results. Current Coumadin dose 5mg. [nures],LPN | [('Coumadin dose 5mg', 'medication')] | [('coumadin', 'medication'), ('5mg.', 'medication')] | [('Coumadin', 'medication'), ('5mg', 'medication')] |
| 5. | just received word [resident] is coming today, they are arranging transport now. also expecting [resident] at anytime | - | - | [('resident', 'what_matters'), ('they are arranging transport', 'what_matters')] |

**Model 1: Machine Learning with count 4Ms**


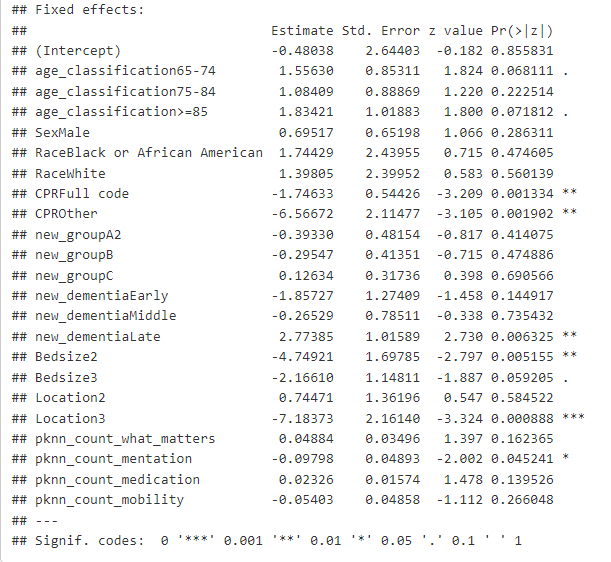


## Estimate Std. Error z value Pr(>|z|)

## (Intercept) -0.48038 2.64403 -0.182 0.855831

## age_classification65-74 1.55630 0.85311 1.824 0.068111 .

## age_classification75-84 1.08409 0.88869 1.220 0.222514

## age_classification>=85 1.83421 1.01883 1.800 0.071812 .

## SexMale 0.69517 0.65198 1.066 0.286311

## RaceBlack or African American 1.74429 2.43955 0.715 0.474605

## RaceWhite 1.39805 2.39952 0.583 0.560139

## CPRFull code -1.74633 0.54426 -3.209 0.001334 **

## CPROther -6.56672 2.11477 -3.105 0.001902 **

## new_groupA2 -0.39330 0.48154 -0.817 0.414075

## new_groupB -0.29547 0.41351 -0.715 0.474886

## new_groupC 0.12634 0.31736 0.398 0.690566

## new_dementiaEarly -1.85727 1.27409 -1.458 0.144917

## new_dementiaMiddle -0.26529 0.78511 -0.338 0.735432

## new_dementiaLate 2.77385 1.01589 2.730 0.006325 **

## Bedsize2 -4.74921 1.69785 -2.797 0.005155 **

## Bedsize3 -2.16610 1.14811 -1.887 0.059205 .

## Location2 0.74471 1.36196 0.547 0.584522

## Location3 -7.18373 2.16140 -3.324 0.000888 ***

## pknn_count_what_matters 0.04884 0.03496 1.397 0.162365

## pknn_count_mentation -0.09798 0.04893 -2.002 0.045241 *

## pknn_count_medication 0.02326 0.01574 1.478 0.139526

## pknn_count_mobility -0.05403 0.04858 -1.112 0.266048

**Model 2: LLM with count 4Ms**


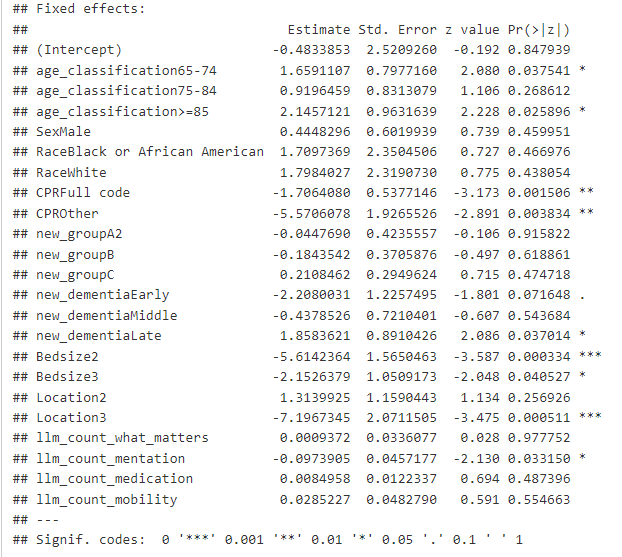


## Estimate Std. Error z value Pr(>|z|)

## (Intercept) -0.4833853 2.5209260 -0.192 0.847939

## age_classification65-74 1.6591107 0.7977160 2.080 0.037541 *

## age_classification75-84 0.9196459 0.8313079 1.106 0.268612

## age_classification>=85 2.1457121 0.9631639 2.228 0.025896 *

## SexMale 0.4448296 0.6019939 0.739 0.459951

## RaceBlack or African American 1.7097369 2.3504506 0.727 0.466976

## RaceWhite 1.7984027 2.3190730 0.775 0.438054

## CPRFull code -1.7064080 0.5377146 -3.173 0.001506 **

## CPROther -5.5706078 1.9265526 -2.891 0.003834 **

## new_groupA2 -0.0447690 0.4235557 -0.106 0.915822

## new_groupB -0.1843542 0.3705876 -0.497 0.618861

## new_groupC 0.2108462 0.2949624 0.715 0.474718

## new_dementiaEarly -2.2080031 1.2257495 -1.801 0.071648 .

## new_dementiaMiddle -0.4378526 0.7210401 -0.607 0.543684

## new_dementiaLate 1.8583621 0.8910426 2.086 0.037014 *

## Bedsize2 -5.6142364 1.5650463 -3.587 0.000334 ***

## Bedsize3 -2.1526379 1.0509173 -2.048 0.040527 *

## Location2 1.3139925 1.1590443 1.134 0.256926

## Location3 -7.1967345 2.0711505 -3.475 0.000511 ***

## llm_count_what_matters 0.0009372 0.0336077 0.028 0.977752

## llm_count_mentation -0.0973905 0.0457177 -2.130 0.033150 *

## llm_count_medication 0.0084958 0.0122337 0.694 0.487396

## llm_count_mobility 0.0285227 0.0482790 0.591 0.554663

**Model 3: Machine Learning with normalized 4Ms**


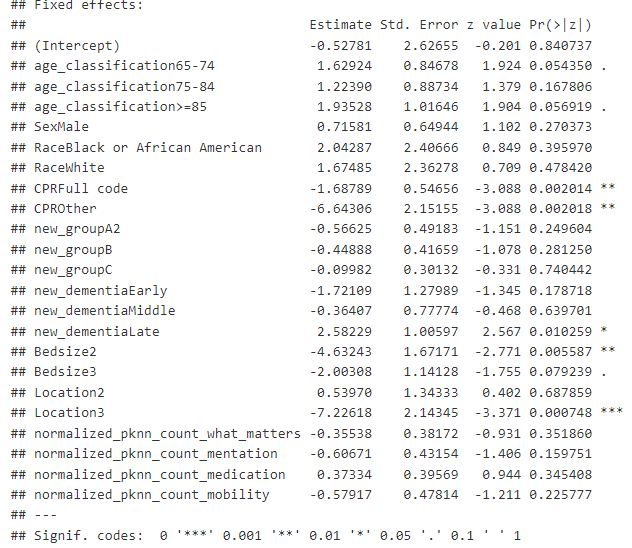


## Estimate Std. Error z value Pr(>|z|)

## (Intercept) -0.52781 2.62655 -0.201 0.840737

## age_classification65-74 1.62924 0.84678 1.924 0.054350 .

## age_classification75-84 1.22390 0.88734 1.379 0.167806

## age_classification>=85 1.93528 1.01646 1.904 0.056919 .

## SexMale 0.71581 0.64944 1.102 0.270373

## RaceBlack or African American 2.04287 2.40666 0.849 0.395970

## RaceWhite 1.67485 2.36278 0.709 0.478420

## CPRFull code -1.68789 0.54656 -3.088 0.002014 **

## CPROther -6.64306 2.15155 -3.088 0.002018 **

## new_groupA2 -0.56625 0.49183 -1.151 0.249604

## new_groupB -0.44888 0.41659 -1.078 0.281250

## new_groupC -0.09982 0.30132 -0.331 0.740442

## new_dementiaEarly -1.72109 1.27989 -1.345 0.178718

## new_dementiaMiddle -0.36407 0.77774 -0.468 0.639701

## new_dementiaLate 2.58229 1.00597 2.567 0.010259 *

## Bedsize2 -4.63243 1.67171 -2.771 0.005587 **

## Bedsize3 -2.00308 1.14128 -1.755 0.079239 .

## Location2 0.53970 1.34333 0.402 0.687859

## Location3 -7.22618 2.14345 -3.371 0.000748 ***

## normalized_pknn_count_what_matters -0.35538 0.38172 -0.931 0.351860

## normalized_pknn_count_mentation -0.60671 0.43154 -1.406 0.159751

## normalized_pknn_count_medication 0.37334 0.39569 0.944 0.345408

## normalized_pknn_count_mobility -0.57917 0.47814 -1.211 0.225777

**Model 4: LLM with normalized 4Ms**


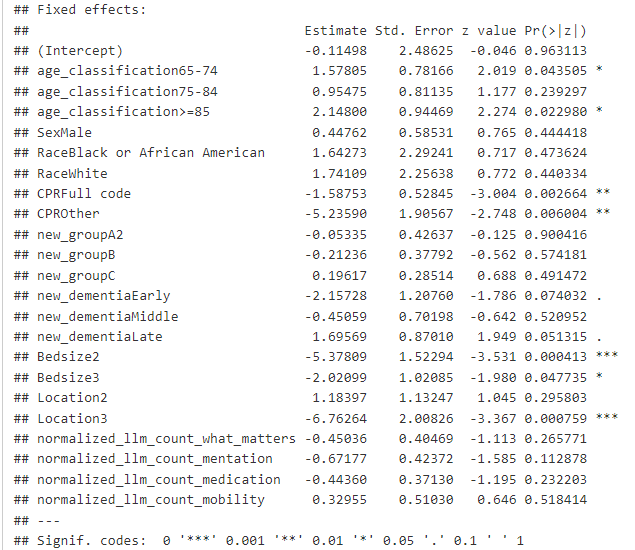


## Fixed effects:

## Estimate Std. Error z value Pr(>|z|)

## (Intercept) -0.11498 2.48625 -0.046 0.963113

## age_classification65-74 1.57805 0.78166 2.019 0.043505 *

## age_classification75-84 0.95475 0.81135 1.177 0.239297

## age_classification>=85 2.14800 0.94469 2.274 0.022980 *

## SexMale 0.44762 0.58531 0.765 0.444418

## RaceBlack or African American 1.64273 2.29241 0.717 0.473624

## RaceWhite 1.74109 2.25638 0.772 0.440334

## CPRFull code -1.58753 0.52845 -3.004 0.002664 **

## CPROther -5.23590 1.90567 -2.748 0.006004 **

## new_groupA2 -0.05335 0.42637 -0.125 0.900416

## new_groupB -0.21236 0.37792 -0.562 0.574181

## new_groupC 0.19617 0.28514 0.688 0.491472

## new_dementiaEarly -2.15728 1.20760 -1.786 0.074032 .

## new_dementiaMiddle -0.45059 0.70198 -0.642 0.520952

## new_dementiaLate 1.69569 0.87010 1.949 0.051315 .

## Bedsize2 -5.37809 1.52294 -3.531 0.000413 ***

## Bedsize3 -2.02099 1.02085 -1.980 0.047735 *

## Location2 1.18397 1.13247 1.045 0.295803

## Location3 -6.76264 2.00826 -3.367 0.000759 ***

## normalized_llm_count_what_matters -0.45036 0.40469 -1.113 0.265771

## normalized_llm_count_mentation -0.67177 0.42372 -1.585 0.112878

## normalized_llm_count_medication -0.44360 0.37130 -1.195 0.232203

## normalized_llm_count_mobility 0.32955 0.51030 0.646 0.518414

**AIC comparisons**


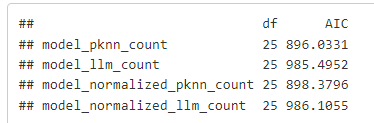


**LLM Prompt**

System Prompt:

The four components of the 4M Framework are:

M1 = What Matters: Understanding and acting on the specific health goals and care preferences of the older adult.

- Examples of M1 Include:

- Discussions about code status, DNR orders, and advanced directives.

- Words or phrases related to palliative care, hospice, or end-of-life care.

- Inclusion of family and caregivers in decision-making and care planning.

- Discussions about quality of life, values, and preferences.

- Aligning treatment and care plans with the older adult's goals and preferences.

M2 = Medication: Ensuring that medications do not interfere with the other aspects of the 4M Framework and are optimized for the older adult's health.

- Examples of M2 Include:

- Medication reconciliation and review.

- Deprescribing or reducing the number of medications.

- Addressing polypharmacy and potential drug interactions.

- Ensuring appropriate medication use and adherence.

- Monitoring for side effects and adverse drug reactions.

M3 = Mentation: Addressing mental health, including cognitive function, depression, and delirium.

- Examples of M3 Include:

- Screening for cognitive impairment and dementia.

- Assessing and managing depression and anxiety.

- Preventing, identifying, and managing delirium.

- Addressing sleep disorders and other mental health issues.

- Promoting brain health and cognitive function.

M4 = Mobility: Promoting physical activity and preventing falls to maintain or improve function.

- Examples of M4 Include:

- Assessing and addressing gait and balance issues.

- Preventing falls and fall-related injuries.

- Encouraging physical activity and exercise.

- Promoting mobility and independence.

- Addressing barriers to physical activity and mobility.

Given this background, please extract and categorize concepts related to the 4M Framework from the following unstructured text messages. For each message, identify and label the relevant concepts under the appropriate "M" category in json format.

Do not include names or any personal information in the reference that can identify the individuals in the message, instead use resident/family or exclude any personal information.

Some messages are not applicable to the 4M framework, in which case you should return an empty string for each concept.

These include greetings, salutations, expressions of gratitude, emojies, out-of-office messages, adding or removing people from the etc.

There are also messages that are very short (sometimes just a number or a letter) and do not contain any relevant information. In these cases, you should return an empty string for each concept.

**Extraction instructions and validation:**

class Classification(BaseModel):

M1_reasoning: str = Field(description="""Provide a brief explanation of why any words or phrases in this text belong to the What Matters concept""")

M1_ref: List[str] = Field(description="""

Make a list of words or phrases that align with the 4M concept - What Matters extracted from this text.

""")

M2_reasoning: str = Field(description="""Provide a brief explanation of why any words or phrases in this text belong to the Medication concept""")

M2_ref: List[str] = Field(description="""

Make a list of words or phrases that aling with the 4M concept - Medication extracted from this text.

""")

M3_reasoning: str = Field(description="""Provide a brief explanation of why any words or phrases in this text belong to the Mentation concept""")

M3_ref: List[str] = Field(description="""

Make a list of words or phrases that align with the 4M concept - Mentation extracted from this text.

""")

M4_reasoning: str = Field(description="""Provide a brief explanation of why any words or phrases in this text belong to the Mobility concept""")

M4_ref: List[str] = Field(description="""

Make a list of words or phrases that align with the 4M concept - Mobility extracted from this text.

""")

pydantic_parser = PydanticOutputParser(pydantic_object=Classification)

format_instructions = pydantic_parser.get_format_instructions()

template_string = """ You are an expert AI specialized with the classification and categorization of unstructured text messages sent to and from clinicians.

Your task is to categorize text messages into the 4M frameowrk of Age-Friendly Health Systems: What Matters, Medication, Mentation, and Mobility.

Here is some background information:

{system_instructions}

Take the message below and identify words and phrases of the the 4M framework.

Here is the message:

"{text}"

You must follow the instructions below to format your response exactly as presented in JSON format.

```

{format_instructions}

```
